# HDAC6, modulated by miR-206, promotes endometrial cancer progression through the PTEN/AKT/mTOR pathway

**DOI:** 10.1038/s41598-020-60271-4

**Published:** 2020-02-27

**Authors:** Yawen Zheng, Xiaohui Yang, Chunyan Wang, Shuo Zhang, Zhiling Wang, Meng Li, Yuanjian Wang, Xiaojie Wang, Xingsheng Yang

**Affiliations:** 1 Department of Obstetrics and Gynecology, Qilu Hospital of Shandong University, Jinan, Shandong China; 2 West China School of Medicine, Sichuan University, Chengdu, Sichuan China; 3 Department of dermatology, Peking University People’s Hospital, Beijing, China

**Keywords:** Endometrial cancer, Oncogenes

## Abstract

Endometrial cancer (EC) is the sixth most common cancer in women. Since early EC has a good prognosis, identifying methods for early diagnosis is valuable. Here, we aimed to study the role of HDAC6, which has been indicated important in many kinds of cancers, in EC diagnosis and therapy. First, the expression levels of HDAC6 in EC tissues and cells were measured by qRT-PCR and Western blotting, and through bioinformatics and dual luciferase assays, HDAC6 was found to be a direct target of miR-206. Then, CCK-8, colony formation, wound healing, and Transwell assays were performed; these results indicated that HDAC6 promoted EC cell proliferation, metastasis and invasion, while miR-206 produced the opposite effects. In addition, rescue assays verified that the effect of miR-206 could be reversed by HDAC6, and global gene expression analysis confirmed the relationship between miR-206 and HDAC6. Finally, we measured the levels of PTEN, p-AKT and p-mTOR and other key molecules and speculated that miR-206 might target HDAC6 to suppress EC progression via the PTEN/AKT/mTOR pathway. In conclusion, downregulation of miR-206 and upregulation of HDAC6 in EC may predict poor prognosis, and as the target gene of miR-206, HDAC achieves its carcinogenic effect through the PTEN/AKT/mTOR pathway.

## Introduction

Endometrial cancer (EC) is the sixth most general female cancer and the 15^th^ most common cancer overall^1^. Over 380,000 new cases were diagnosed in 2018^1^, and estimates indicated that 61880 people would be newly diagnosed and 12160 people would die of EC in the USA in 2019^2^. Conventionally, EC has been classified into two universal subtypes: type I (endometrioid) and type II (non-endometrioid). Characterized by an oestrogen-related, low-grade phenotype and good prognosis, type I is the most common (85–90%)^3^. However, for both types, the earlier the diagnosis and intervention, the better are the long-term outcomes. Thus, determining the molecular mechanism of EC development and progression would aid the design of diagnostic and therapeutic approaches to improve survival.

Recently, a class of endogenous noncoding small RNAs, microRNAs (miRNAs) have become a research focus^4^. Generally incorporating approximately 22 nucleotides, miRNAs regulate the expression of gene by binding to the 3′-untranslated region (UTR) of target mRNAs^5^. MiRNAs have been proven to exert important effects on many cellular processes, such as cell differentiation, proliferation and apoptosis^6–8^. In a recent meta-analysis, the expression levels of 261 miRNAs in EC were collected from literature reviews and original articles, and the results suggested that miRNAs analysis deserved a role in the evaluation of prognostic factors and diagnostic markers in the management of EC patients^9^; specifically, upregulation of miR-182, miR-183, miR-200a, miR-200b, and miR-205 and downregulation of miR-152 were the most frequently implicated miRNA alterations in EC^10–12^. MiR-206 has been shown to be markedly downregulated in many cancers, such as lung cancer, breast cancer, rhabdomyosarcoma and head and neck squamous cell carcinoma^13–16^, but its function in EC is unclear. Therefore, we aimed to elucidate the molecular mechanisms of miR-206 in EC.

Histone deacetylase (HDAC) enzymes are divided into four classes—class I (HDAC1, 2, 3, and 8), class II (HDAC4, 5, 6, 7, 9, and 10), class III (SIRT1–7), and class IV (HDAC11)—and remove acetyl groups (O=C-CH3) from ε-N-acetyl lysine amino acids on histones, allowing the histones to wrap DNA more tightly^17^. Histone deacetylase 6 (HDAC6) deacetylates numerous substrates to regulate protein translocation and degradation as well as cell shape modification and migration; moreover, unlike other HDACs, HDAC6 performs its functions in the cytoplasm primarily^18^. Several studies have shown that HDAC6 expression is associated with oncogene mutations and tumour formation in several human cancers, including ovarian and breast cancers^19,20^.

Here, we hypothesized that HDAC6 could contribute to the progression of EC and may thus be a potential diagnostic marker and a promising prognostic predictor in patients with EC. We investigated HDAC6 expression in EC specimens and cell lines and validated its function of promoting proliferation and migration *in vitro*. Furthermore, we found that miR-206 might directly bind to the 3′-UTR of HDAC6 and posttranscriptionally downregulate its expression. Then, we evaluated the role of miR-206 in tumorigenesis and revealed that HDAC6 can reverse the effect of miR-206 *in vitro*. Finally, we demonstrated that miR-206 prevents cancer progression in EC by downregulating HDAC6 via the PTEN/AKT/mTOR pathway. Our results suggest that miR-206 and HDAC6 play critical roles in EC development and may be innovative diagnostic markers and therapeutic targets for EC.

## Materials and Methods

### Bioinformatic analysis of clinical data

The EC data set was obtained from The Cancer Genome Atlas (TCGA) database, and the overall survival of patients was assessed with data from the Human Protein Atlas (https://www.proteinatlas.org). UALCAN (http://ualcan.path.uab.edu) was used for clinicopathological analysis of HDAC6. A *P*-value of <0.05 was considered statistically significant.

### Human EC specimens

Paraffin-embedded tissue samples were acquired from 36 EC patients and 8 patients with dysfunctional uterine bleeding and normal curettage results between January 2010 and December 2013 at Qilu Hospital of Shandong University (Jinan, China). In addition, fresh EC and matched adjacent nontumour tissues from 46 patients were obtained instantly after surgical excision from patients in the Department of Obstetrics and Gynecology, Qilu Hospital of Shandong University, between January 2017 and December 2018. These samples were immediately frozen in liquid nitrogen and subsequently stored at −80 °C. All EC samples were endometrioid adenocarcinoma, no patient received treatment except for surgical excision. The Medical Ethics Committee of Qilu Hospital reviewed and approved this study (KYLL‐2018‐372). Before surgery, all participants signed written informed consent forms.

### Immunohistochemical (IHC) analysis

Paraffin-embedded tissue sections were deparaffinized and rehydrated through a series of graded ethanol concentrations and distilled water. After washing with phosphate-buffered saline (PBS), sections were blocked using reagent 1 (PV-9001; ZSGB-BIO, China) for 30 min at room temperature. Subsequently, tissues were incubated with an anti-HDAC6 antibody (ab133493; Abcam, 1:300) or PBS, which was used as the negative control, overnight at 4 °C. After washing in PBS, sections were incubated independently with reagents 2 and 3 (PV-9001; ZSGB-BIO, China). Finally, 3,3′-diaminobenzidine and 10% Mayer’s haematoxylin were used for visualization and nuclear localization.

### Total RNA isolation and quantitative real-time PCR (qRT-PCR)

Total RNA was isolated from tissues or cells with TRIzol reagent (15596018; Invitrogen, Waltham, MA). A NanoDrop One Microvolume UV-Vis Spectrophotometer (Thermo Fisher Scientific Inc., Waltham, MA) was used to measure the mRNA concentrations. Then, cDNA was obtained with ReverTra Ace qPCR RT Master Mix with genomic DNA (gDNA) Remover (FSQ-301; Toyobo, Japan). SYBR Green Real-time PCR Master Mix (QPK-201; Toyobo, Japan) was used to quantify the relative miRNA and mRNA expression levels. The levels of mRNA and miR-206 were normalized to those of glyceraldehyde-3-phosphate dehydrogenase (GAPDH) and U6, respectively. The primers used were as follows: human HDAC6, forward primer (5′-CAACTGAGACCGTGGAGAG-3′) and reverse primer (5′-CCTGTGCGAGACTGTAGC-3′); human GAPDH, forward primer (5′-GCACCGTCAAGGCTGAGAAC-3′) and reverse primer (5′-TGGTGAAGACGCCAGTGGA-3′); miR-206, 5′-TGGAATGTAAGGA AGTGTGTGG-3′; and hsRNA-U6, 5′-CGCTTCGGCAGCA CATATACTA-3′. The 2^−ΔΔCt^ method was used to determine the relative quantification.

### Cell lines and cell culture

Three human EC cell lines, Ishikawa, AN3C and RL95, were obtained from the Key Laboratory of Gynecologic Oncology of Shandong Province. The HEC-1-A and immortalized endometrial fibroblast cell lines were purchased from Icellbioscience (Shanghai, China). AN3C, RL95 and HEC-1-A cells were cultured in DMEM/F12 medium (SH30023.01; HyClone, USA), while Ishikawa and immortalized endometrial fibroblast cells were cultured in RPMI 1640 medium (SH30809.01; HyClone, USA). In addition, all media were supplemented with 10% foetal bovine serum (FBS; 04-001-1 A; Biological Industries, Israel) and 1% penicillin-streptomycin (P1400; Solarbio, China), and all cells were cultured at 37 °C in 5% CO_2_.

### Lentiviral transduction

Lentiviruses carrying vectors expressing shRNA targeting human HDAC6, the amplified human HDAC6 sequence or their corresponding negative control vectors were designed and synthesized by GeneChem (Shanghai, China). Lentiviruses carrying miR-206 mimic, inhibitor and scrambled negative control RNA sequences were also designed and synthesized by GeneChem (Shanghai, China). The sequences carried by the lentiviruses were as follows: HDAC6 siRNA (5′-CACTTCGAAGCGAAATATT-3′), HDAC6 siRNA-NC (5′-TTCTCCGAACGTGTCACGT-3′), miR-206 mimic (sense (S): 5′-UGGAAUGUAAGGAAGUGUGUGG-3′; antisense (A): 5′-ACACACUUCCUUACAUUCCAUU-3′), miR-206 mimic negative control (S: 5′-UUCUCCGAACGUGUCACGUTT-3′; A: 5′-ACGUGACACGUUCGGAGAATT-3′), miR-206 inhibitor (5′-CCACACACUUCCUUACAUUCCA-3′), and miR-206 inhibitor negative control (5′-CAGUACUUUUGUGUAGUACAA-3′). After infection with the viral suspension for 24 h, complete medium containing 1 µg/ml puromycin was used to culture cells for one week to screen for stable knockdown (KD) and overexpression (OE) clones. The names of these clones are as follows: Ishikawa, Ishikawa^miR206_NC^, Ishikawa^miR206−^, Ishikawa^miR206+^, Ishikawa^HDAC6+^, Ishikawa^HDAC6+_NC^, Ishikawa^siHDAC6^, Ishikawa^siHDAC6_NC^, Ishikawa^miR206+/HDAC6+^, and Ishikawa^miR206−/siHDAC6^ (and similar for the AN3C cell lines). The stable knockdown and overexpression clones were confirmed by qRT-PCR and Western blot analyses.

### Dual luciferase assay

TargetScan (http://www.targetscan.org) analysis showed that the 3′-UTR of HDAC6 might contain a miR-206 binding site. GeneChem (Shanghai, China) helped to chemically synthesize the wild-type or mutant 3′-UTR of HDAC6 containing the putative binding sites for miR-206, and cloned them into the psicheck2 dual luciferase vector. Twenty-four hours after transfection into Ishikawa cells, luciferase activity was analysed with a DualGlo luciferase assay kit (Promega).

### Cell proliferation

A cell Counting Kit-8 (CCK-8; APExBIO, USA) was used for cell proliferation assays. Cells were seeded into a 96-well plate at a density of 5 × 10^3^ cells/well in quintuplicate wells. At 4, 28, 52, 76 and 100 h after culture, 10 μl of CCK-8 solution was added to each well, and after another 1.5 h of incubation at 37 °C, the absorbance of cells was measured at a wavelength of 450 nm for calculation of the optical density (OD) values.

### Colony formation assay

Cells in each group were seeded in a 6-well plate at a density of 800 cells/well and cultured for 12 days. Then, cells were fixed with 100% methanol for 25 min and stained with 0.1% crystal violet for 30 min. Finally, colonies were counted under an inverted microscope.

### Wound healing assay

Cells were plated in 6-well plates and incubated to almost complete confluence, and a 10 μl pipette tip was then used to scratch the cell layer. Then, cells were incubated in serum-free medium. The gap widths immediately after scratching (0 h) and at 24–48 h after scratching were measured and imaged under an inverted microscope.

### Transwell migration and invasion assay

Cell migration and invasion capabilities were detected through Transwell assays (3422; Corning USA). In brief, cells were collected and suspended in 200 μl of serum-free medium. For the migration assay, 8 × 10^4^ cells in serum-free medium were added to the upper chambers. For the invasion assay, the Transwell chambers were precoated with 60 μl of Matrigel (356234; Corning, USA; 1:7 dilution), and after culturing for 1 h at 37 °C to allow solidification of the Matrigel, 8 × 10^4^ cells in serum-free medium were added to the upper chambers. The lower chambers were filled with 600 μl of complete medium. After incubation for 24 h at 37 °C and 5% CO_2_, the invaded cells were fixed with methanol for 25 min and were then stained with 0.1% crystal violet for 30 min. Then, the invaded cells were counted in at least five random fields under a light microscope (200×).

### Western blot analysis

Total protein was extracted from cells or tissues with a Total Protein Extraction Kit (BestBio, China). Equivalent amounts of proteins were isolated by 10% SDS-PAGE and were then transferred to a PVDF membrane. A solution of 5% fat-free milk in Tris-buffered saline (TBS) was used for blocking. After blocking for 1 h at room temperature, membranes were incubated first with primary antibodies against HDAC6 (ab133493; Abcam, 1:1000), PTEN (AF6351; Affinity, 1:1000), AKT (4685; Cell Signaling Technology, 1:1000), p-AKT (4060; Cell Signaling Technology, 1:1000), mTOR (GB11405; Servicebio, 1:1000), p-mTOR (5536; Cell Signaling Technology, 1:1000), and GAPDH (2118) at 4 °C overnight, and then with a goat anti-rabbit IgG secondary antibody (7074; Cell Signaling Technology, 1:2000) for 1.5 h at room temperature. Enhanced chemiluminescence (ECL) reagent (Thermo) was used for visualization. GAPDH was used as the internal control.

### RNA sequencing analysis

Total cellular RNA was extracted from three groups of cells (AN3C^miR206−^, AN3C^miR206+^ and AN3C^miR206_NC^) for mRNA sequencing (mRNA-seq). A kaiaoK5500 Spectrophotometer (Kaiao, Beijing, China) was used to assess RNA purity, and the RNA Nano 6000 Assay Kit for the Bioanalyzer 2100 system (Agilent Technologies, USA) was used to assess RNA integrity and concentration. Libraries were prepared and were then sequenced using an Illumina HiSeq X10 instrument (Xiuyue Biol, China). Quality control was performed on the raw data were with fastp software, and mapping was conducted using STAR software. The read counts were obtained with HTseq, and the significantly differentially expressed genes were identified by the DESeq. 2 package with an adjusted *P*-value cutoff of 0.05. Enrichment analysis was performed with the clusterprofiler package.

### Statistical analysis

All experiments in the study were performed in triplicate. Quantitative data are presented as the mean values ± standard deviations (SDs). Student’s t-test or the Wilcoxon matched pairs test was used to determine the significance of the results, whereas Fisher’s test was utilized to compare categorical data. Statistical analysis was performed using SPSS 19.0 (SPSS Inc., USA); heat maps were generated and KEGG pathway analyses were performed using R version 3.6.1 (www.r-project.org). The significance levels of the data are denoted by * symbols as follows: *P < 0.05; **P < 0.01; ***P < 0.001; and ****P < 0.0001. *P* < 0.05 was considered statistically significant.

All procedures were performed in strict conformity with the relevant guidelines and regulations.

## Results

### HDAC6 is upregulated in EC tissues and cell lines

**Figure 1:**
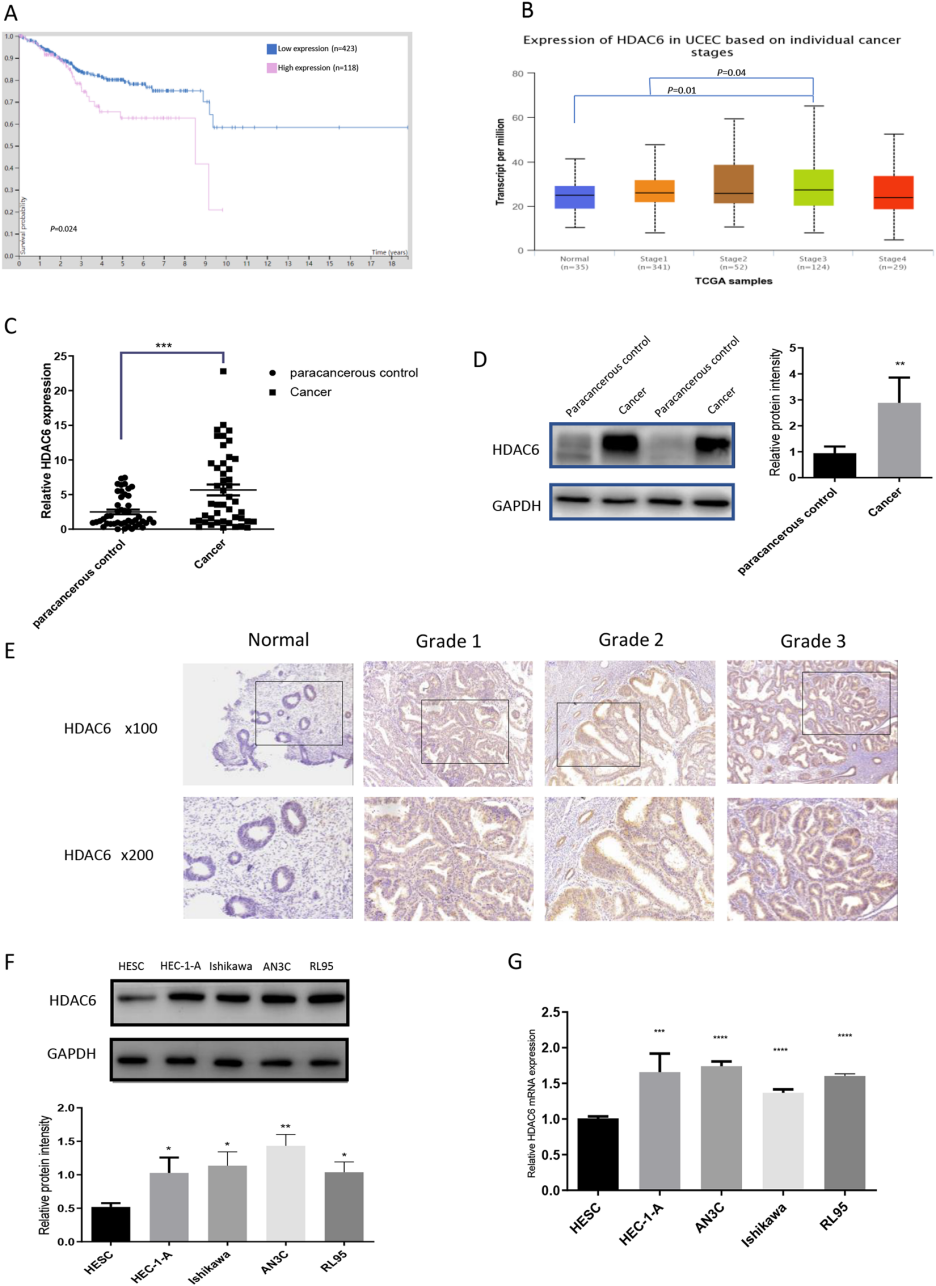
HDAC6 is upregulated in EC tissues and cell lines. (**A**) According to the Human Protein Atlas (https://www.proteinatlas.org), Kaplan-Meier survival curves indicated that high HDAC6 expression was correlated with poorer survival probability. (**B**) HDAC6 expression in different EC stages according to UALCAN (http://ualcan.path.uab.edu/index.html). (**C**) qRT-PCR showed higher HDAC6 expression in EC tissues than in paired adjacent normal tissues (n = 46). (**D**) Western blot analysis showed that cancer tissues expressed more HDAC6 than paracancerous control tissues (n = 46). (**E**) IHC analysis showed the HDAC6 expression levels in paraffin-embedded normal endometrial and EC specimens (100×, 200×). (**F**) Western blot analysis showed the HDAC6 expression levels in four selected EC cell lines—HEC-1-A, Ishikawa, AN3C, and RL95—and in one immortalized endometrial fibroblast cell line. (**G**) qRT-PCR showed HDAC6 mRNA levels in the EC cell lines and immortalized endometrial fibroblast cell line. **P < 0.01, ***P < 0.001, ****P < 0.0001.

To explore the role of HDAC6 in human EC, we extracted its expression data and clinical data from TCGA datasets in the Human Protein Atlas (https://www.proteinatlas.org). Kaplan-Meier survival curves revealed that patients (n = 118) with high HDAC6 expression exhibited poorer survival than those (n = 423) with low HDAC6 expression (*P* = 0.024, Fig. 1A). In addition, analysis of data for cancers of different stages retrieved from UALCAN (http://ualcan.path.uab.edu/index.html) revealed that compared to normal tissue and stage I tissue, stage III tissue exhibited increased HDAC6 expression (*P* = 0.01 and 0.04, respectively; Fig. 1B).

Then, we compared HDAC6 levels in EC tissues and paired paracancerous tissues from 46 patients. The results of qRT-PCR (*P* < 0.001, n = 46, Fig. 1C) and Western blot (*P* < 0.01, n = 46, Fig. 1D, Supplementary Fig. 1A) analyses revealed significant upregulation of HDAC6 in cancer tissues compared with paracancerous controls. Furthermore, IHC analysis of 36 paraffin-embedded EC tissues and 8 normal endometrial tissues showed that HDAC6 was expressed mainly in the cytoplasm and that the staining intensity increased with increasing tumour grade (Fig. 1E).

HDAC6 expression levels were further assessed in four EC cell lines—HEC-1-A, Ishikawa, AN3C, and RL95—and one immortalized endometrial fibroblast cell line. Both qRT-PCR and Western blotting showed increased expression of HDAC6 in the EC cell lines, with the strongest upregulation in AN3C cells, which are derived from a poorly differentiated human endometrial adenocarcinoma (Fig. 1F,G). These results displayed that HDAC6 was upregulated in both EC cell lines and EC tissues, confirming it as a tumour suppressor in EC.

### HDAC6 promotes the proliferation and tumorigenicity of human EC cells

**Figure 2:**
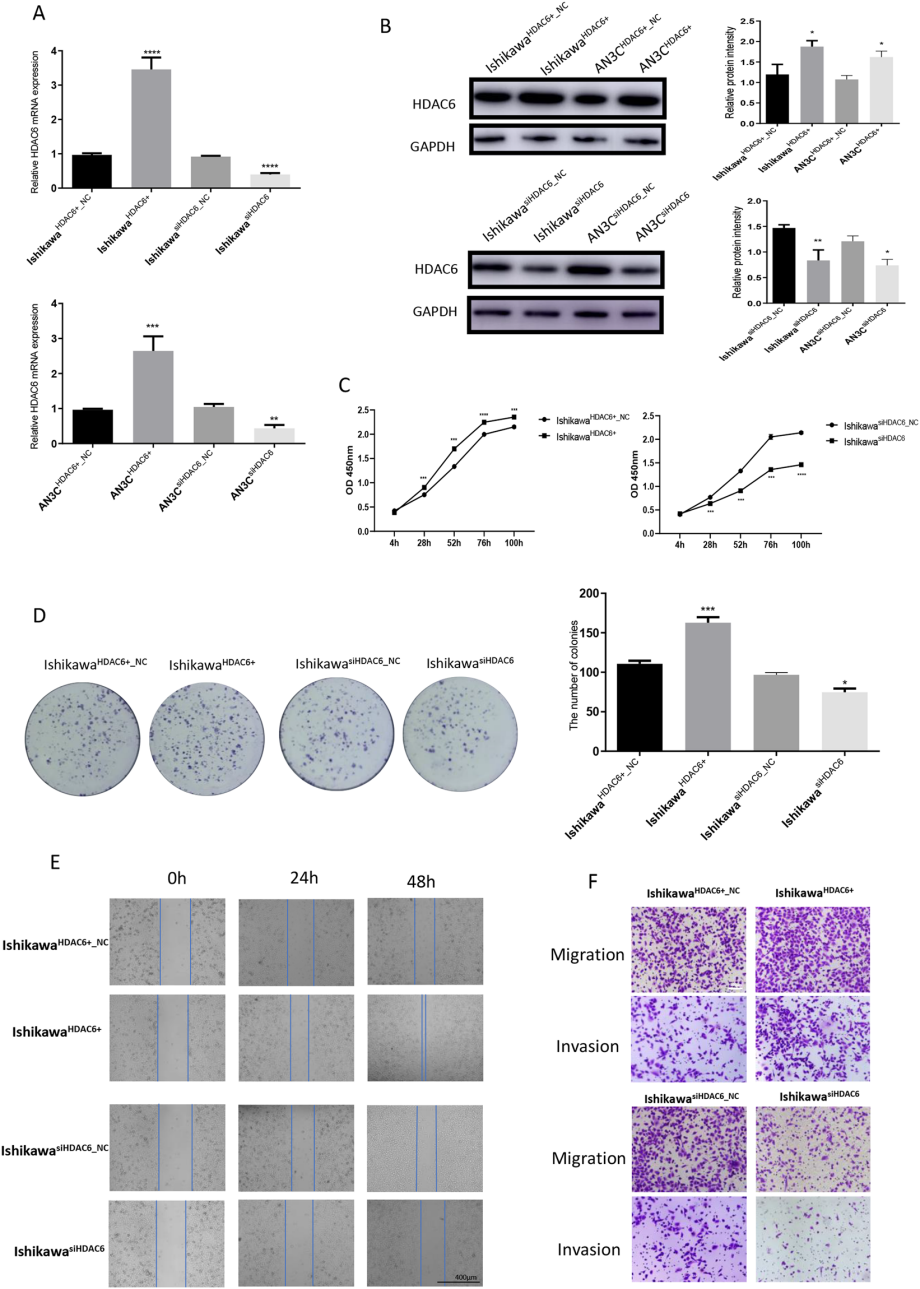
HDAC6 promotes the proliferation and tumorigenicity of human EC cells. (**A**) qRT-PCR showed HDAC6 mRNA levels in Ishikawa and AN3C cells after transfection with generated lentiviruses. (**B**) Western blotting showed HDAC6 protein levels in Ishikawa and AN3C cells after transfection with generated lentiviruses. (**C**) CCK-8 assay after overexpression and knockdown of HDAC6 in Ishikawa cells. (**D**) Colony formation assay after overexpression and knockdown of HDAC6 in Ishikawa cells. (**E**) Wound healing assay after overexpression and knockdown of HDAC6 in Ishikawa cells. Scale bar, 400 μm. (**F**) Transwell migration and invasion assays after overexpression and knockdown of HDAC6 in Ishikawa cells. Scale bar, 100 μm.*P < 0.05, **P < 0.01, ***P < 0.001, ****P < 0.0001.

To explore the biological functions of HDAC6 in EC cells, HDAC6 knockdown and overexpression cell lines were established using lentivirus-transfected Ishikawa cells (derived from a well-differentiated human endometrial adenocarcinoma) and AN3C cells (derived from a poorly differentiated human endometrial adenocarcinoma). Successful establishment of Ishikawa^HDAC6+^, Ishikawa^HDAC6+_NC^, Ishikawa^siHDAC6^, Ishikawa^siHDAC6_NC^, AN3C^HDAC6+^, AN3C^HDAC6+_NC^, AN3C^siHDAC6^ and AN3C^siHDAC6_NC^ cells was confirmed by qRT-PCR (*P* < 0.0001, *P* < 0.001, respectively; Fig. 2A) and Western blotting (*P* < 0.0001, *P* < 0.01, respectively; Fig. 2B) after lentiviral transfection. The role of HDAC6 in tumour cell growth was further validated with CCK-8 assays (Fig. 2C, Supplementary Fig. 1B); these results showed that HDAC6 overexpression promoted proliferation, whereas HDAC6 silencing significantly inhibited proliferation in these two kinds of cell lines. We also performed colony formation assays using these cells (Fig. 2D, Supplementary Fig. 1C). As expected, OE cells exhibited a significantly enhanced colony-forming ability (*P* < 0.0001), and inversely, KD cells exhibited an obviously reduced colony-forming ability (*P* < 0.02). In tumour progression, distant metastasis is a crucial process; thus, we applied a wound healing assay to assess the effect of HDAC6 on EC cell migration. The motility of both Ishikawa and AN3C cells was enhanced following HDAC6 overexpression and inhibited after HDAC6 knockdown (Fig. 2E, Supplementary Fig. 1D). Similar results were obtained in Transwell assays to detect the effects of HDAC6 on the migration and invasion of EC cells—EC cells overexpressing HDAC6 exhibited significantly increased cell migration and invasion capacities, whereas those with knockdown of HDAC6 exhibited markedly reduced migration and invasion (Fig. 2F, Supplementary Fig. 1E). Collectively, these findings demonstrated that HDAC6 promotes the proliferation, migration and invasion of human EC cells.

### HDAC6 is a direct target gene of miR-206

**Figure 3:**
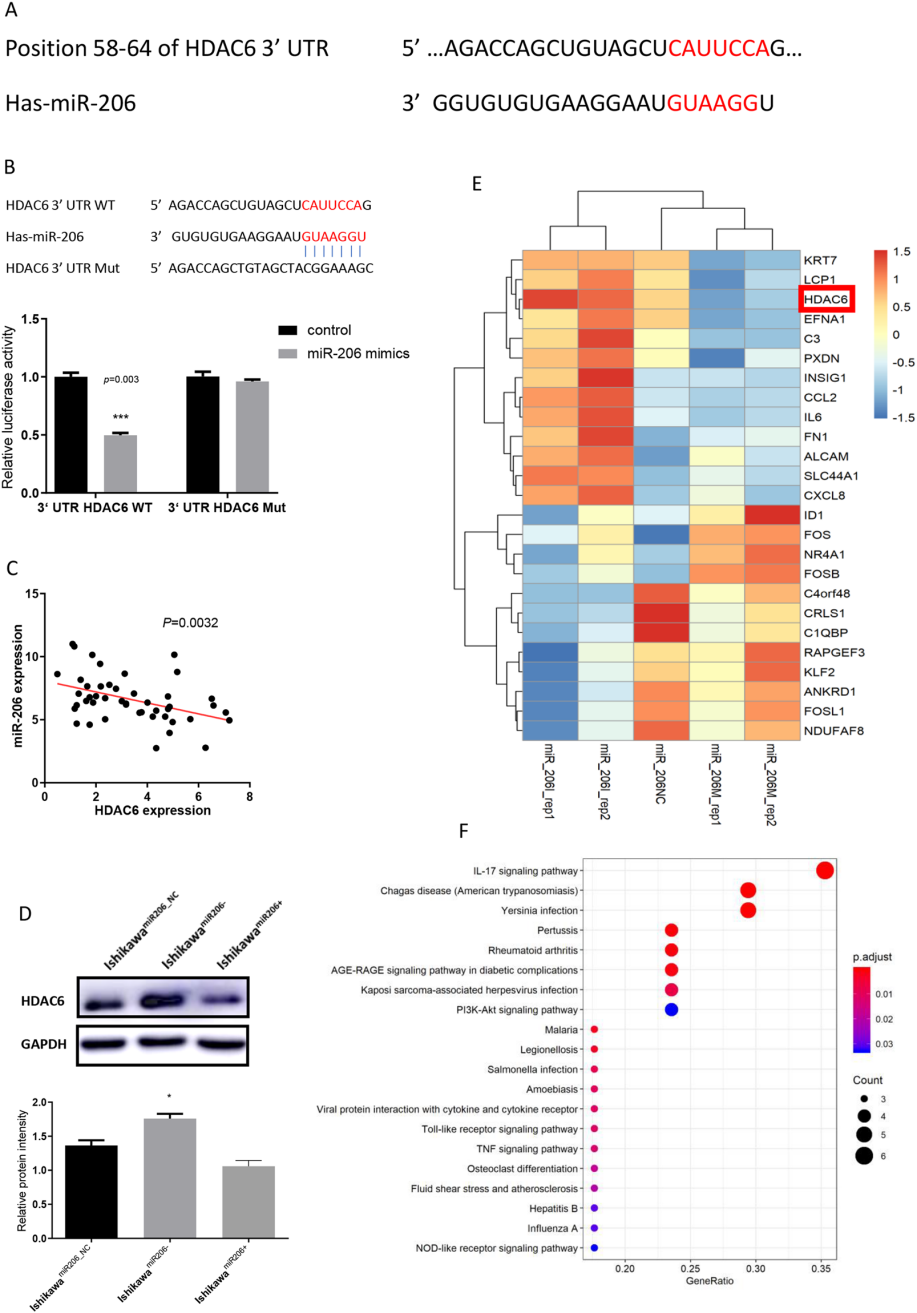
HDAC6 is a direct target gene of miR-206. (**A**) The TargetScan (http://www.targetscan.org/) prediction algorithm identified miR-206 as a potential miRNA targeting the 3′-UTR of HDAC6 mRNA. (**B**) A dual luciferase assay was conducted to verify the prediction. (**C**) The relationship between miR-206 and HDAC6 in EC tissues was shown by Pearson correlation analysis (n = 46). (**D**) Western blotting showed HDAC6 protein levels in Ishikawa cells after transfection with the miR-206 inhibitor and miR-206 mimic. (**E**) Heat map showing the dysregulated genes after miR-206 overexpression and knockdown in AN3C cells. (**F**) KEGG pathway analysis of the top 25 differentially expressed genes. *P < 0.05, ***P < 0.001.

The observed upregulation of HDAC6 in EC tissues and cells prompted us to investigate the mechanism underlying this aberrant expression. We searched potential binding miRNAs of HDAC6 in TargetScan (http://www.targetscan.org/) and found that miR-206 could directly bind the 3′-UTR of HDAC6 mRNA (Fig. 3A). In addition, a luciferase reporter assay was performed to confirm this prediction. Compared with the negative control RNA, miR-206 significantly reduced the luciferase activity of the wild-type HDAC6 3′-UTR (Fig. 3B, *P* = 0.003). Furthermore, in EC tissues, an inverse linear correlation between miR-206 and HDAC6 levels was revealed by Pearson correlation analysis, with R^2^ = 0.1808 and *P* = 0.0032 (Fig. 3C). Subsequent Western blot analyses verified that the miR-206 mimic markedly reduced the expression of HDAC6 in Ishikawa cells, whereas cells transfected with the miR-206 inhibitor showed increased HDAC6 expression (Fig. 3D). Finally, to identify aberrantly expressed genes after overexpression and knockdown of miR-206 in AN3C cells, we conducted a global gene expression analysis (Supplementary Table). The differentially expressed genes were displayed in a heat map and then grouped into gene pathways for KEGG pathway analysis (Fig. 3E,F). HDAC6 expression was higher in EC cells transfected with the miR-206 inhibitor than in EC cells transfected with the miR-206 mimics. These findings implied that miR-206 might directly bind to the 3′-UTR of HDAC6 and posttranscriptionally suppress its expression.

### miR-206 is downregulated in EC tissues and suppresses the proliferation, migration and invasion of endometrial cells *in vitro*

**Figure 4:**
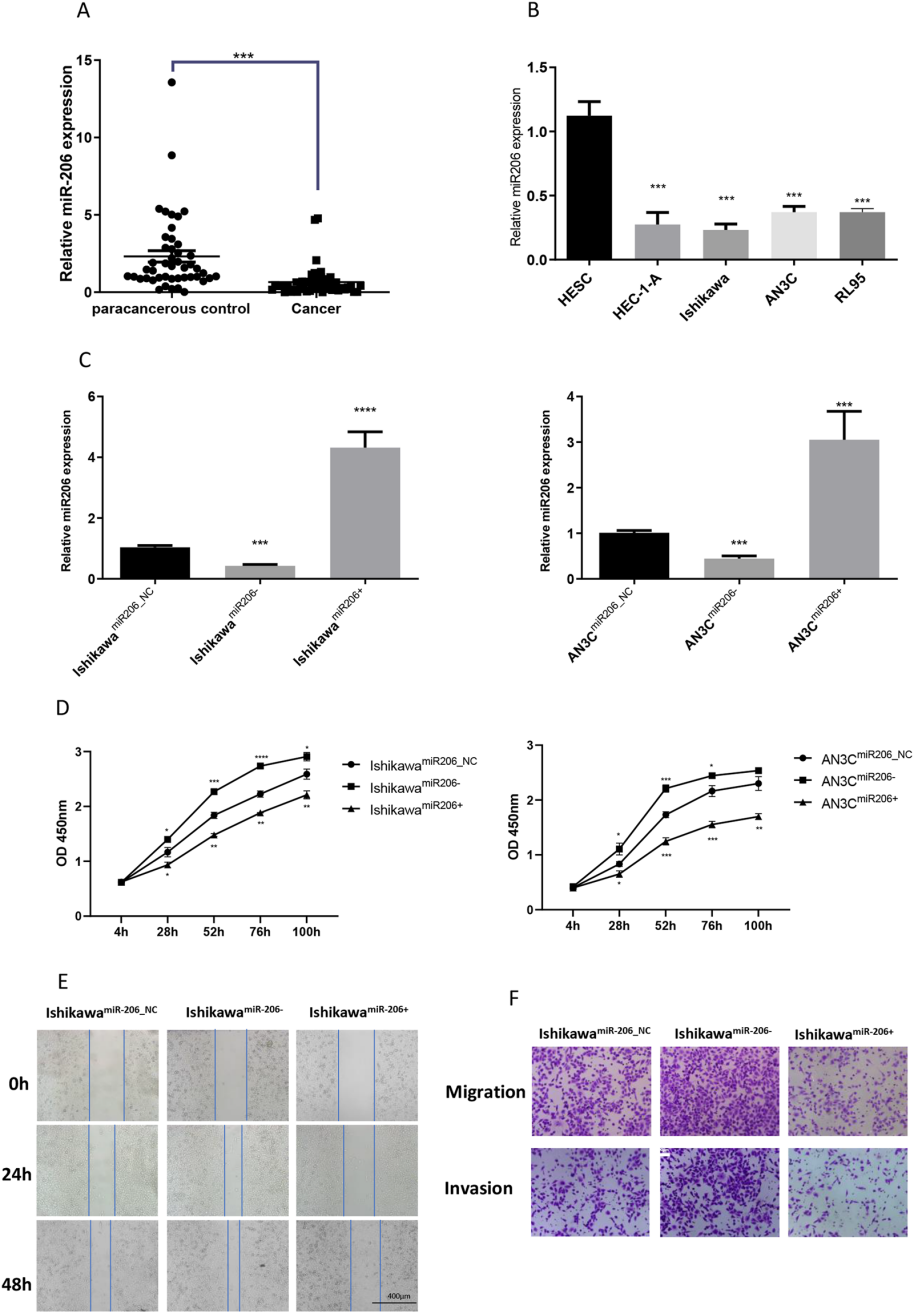
MiR-206 is downregulated in EC tissues and suppresses the proliferation, migration and invasion of endometrial cells *in vitro*. (**A**) qRT-PCR showed miR-206 levels in EC tissues (n = 46). (**B**) qRT-PCR showed miR-206 levels in the EC cell lines and the immortalized endometrial fibroblast cell line. (**C**) qRT-PCR showed miR-206 levels in Ishikawa and AN3C cells after transfection with generated lentiviruses. (**D**) CCK-8 assay of Ishikawa and AN3C cells transfected with the miR-206 inhibitor and miR-206 mimic. (**E**) Scratch wound assay of Ishikawa cells transfected with the miR-206 inhibitor and miR-206 mimic. Scale bar, 400 μm. (**F**) Transwell migration and invasion assays of Ishikawa cells after transfection with the miR-206 inhibitor and miR-206 mimic. Scale bar, 100 μm. *P < 0.05, **P < 0.01, ***P < 0.001, ****P < 0.0001.

To determine whether miR-206 was related to EC development, we then evaluated miR-206 levels in human EC tumour tissues and selected EC cell lines. MiR-206 levels were lower in EC tissues than in the paired paracancerous control tissues (*P* < 0.001, n = 46; Fig. 4A). Similarly, less miR-206 was detected in EC cells than in immortalized endometrial fibroblast cells (*P* < 0.001, Fig. 4B).

Furthermore, using mimics and inhibitors of miR-206, we performed gain- and loss-of-function experiments to explore its role in regulating the progression of EC. The qPCR assay results verified that miR-206 was significantly upregulated in cells transfected with the miR-206 mimic (*P* < 0.0001, Fig. 4C) and significantly downregulated in cells transfected with the miR-206 inhibitor (*P* < 0.001, Fig. 4C). In both the Ishikawa and AN3C cell lines, CCK-8 assays and colony formation assays revealed that compared with the corresponding negative control cells, EC cells exhibited an impaired proliferation capacity after transfection with the miR-206 mimic, whereas miR-206 inhibitor-transfected cells exhibited an enhanced proliferation capacity (Fig. 4D; Supplementary Fig. 2A,B; *P* < 0.01). In addition, the wound healing and Transwell assay results suggested that miR-206 overexpression significantly inhibited but miR-206 knockdown markedly promoted the migration of EC cells (Fig. 4E,F; Supplementary Fig. 2C). In summary, these data implied that miR-206 was downregulated in EC tissues and cells and exhibited negative regulatory effects on EC cell proliferation, migration and invasion *in vitro*.

### HDAC6 can reverse the effect of miR-206 on EC cells

**Figure 5:**
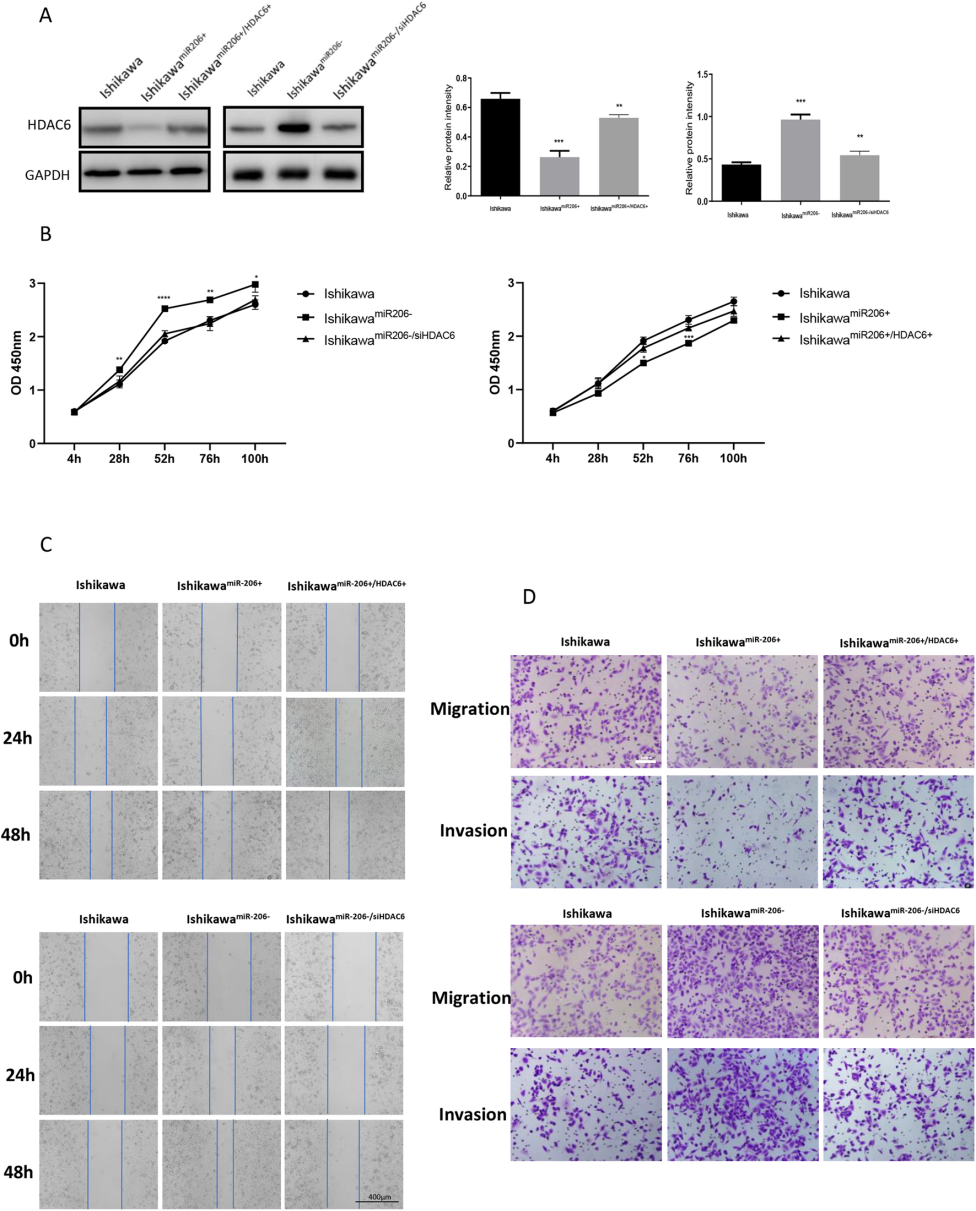
HDAC6 can reverse the effect of miR-206 on EC cells. (**A**) Western blotting showed that HDAC6 protein levels were reversed in Ishikawa cells after co-transfection with the miR-206 mimic and HDAC6 plasmid. (**B**) CCK-8 assay after co-transfection of Ishikawa cells. (**C**) Wound healing assay after co-transfection of Ishikawa cells. Scale bar, 400 μm. (**D**) Transwell migration and invasion assays after co-transfection of Ishikawa cells. Scale bar, 100 μm. ***P < 0.001, ****P < 0.0001.

Considering that HDAC6 might be a direct target of miR-206, we next evaluated whether the effects of miR-206 on EC cells were exerted by targeting HDAC6. First, we measured HDAC6 expression after co-transfecting cells with the miR-206 mimic and either the HDAC6 plasmid or blank vector (Fig. 5A). The decrease in HDAC6 expression induced by the miR-206 mimic was rescued by the HDAC6 vector. In addition, the suppressive effect of miR-206 on proliferation was abolished after co-transfection with the HDAC6 plasmid (Fig. 5B). Furthermore, the wound healing and Transwell assay results showed that the inhibition of the migration and invasion capacities induced by the miR-206 mimic was reversed by HDAC6 overexpression (Fig. 5C,D). In contrast, the increase in HDAC6 expression induced by the miR-206 inhibitor was inhibited by co-transfection of shHDAC6, and the enhancement of the proliferation, migration and invasion capacities induced by the miR-206 inhibitor was reversed by HDAC6 silencing (Fig. 5B–D). Taken together, these results suggested that miR-206 can regulate the biological behaviour of Ishikawa cells at least partially by targeting HDAC6.

### Pathways modulated by HDAC6 in EC cells

The PTEN/AKT/mTOR signalling pathway has been demonstrated to be an important mediator in the development of EC^21^, and KEGG pathway analysis (Fig. 3F) showed that some differentially expressed genes after overexpression and knockdown of miR-206 in Ishikawa cells were in the AKT pathway. Therefore, to clarify the modulatory role of HDAC6 in this critical pathway, we conducted Western blot assays to evaluate the related proteins in this pathway, including PTEN, p-AKT, AKT, p-mTOR and mTOR, in Ishikawa, Ishikawa^HDAC6+^, Ishikawa^HDAC6+_NC^, Ishikawa^siHDAC6^ and Ishikawa^siHDAC6_NC^ cells.

**Figure 6:**
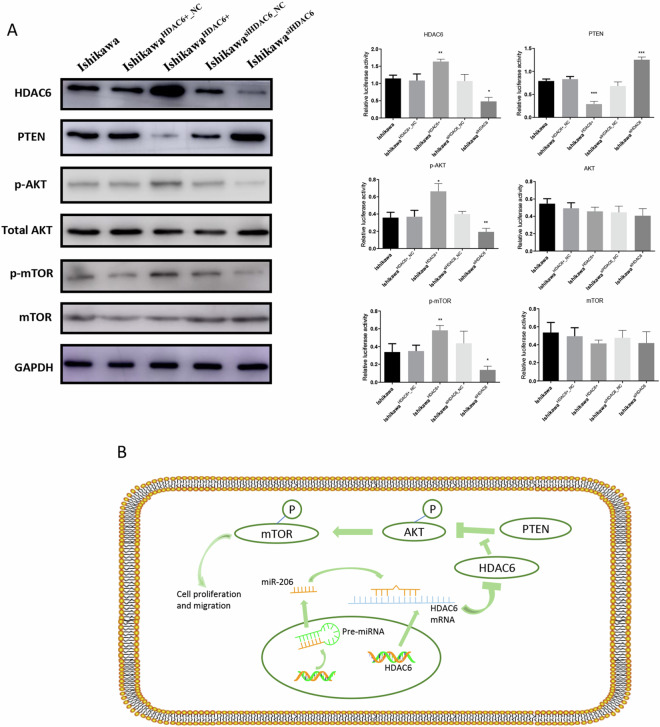
Pathways modulated by HDAC6 in EC cells. (**A**) Western blotting showed the levels of differentially expressed proteins in Ishikawa cells following transfection with generated lentiviruses. (**B**) Putative working model depicting the role of miR-206/HDAC6 in EC progression. *P < 0.05, **P < 0.01, ***P < 0.001.

As shown in Fig. 6A, following HDAC6 overexpression, HDAC6 protein levels were dramatically decreased, and PTEN expression levels were also decreased. Furthermore, p-AKT and p-mTOR levels were increased after HDAC6 overexpression, while no significant changes in total AKT and mTOR levels were observed in the five cell groups. These results indicated that miR-206 might directly bind to the HDAC6 3′-UTR and that HDAC6 could exert its inhibitory effects on EC cell proliferation and metastasis via the HDAC6/PTEN/AKT/mTOR pathway (Fig. 6B).

## Discussion

As a critical mediator of histone acetylation and deacetylation, HDAC6 is expressed in normal tissues, such as the brain, heart, liver, kidney and pancreas^22^, and in many diseases, such as Alzheimer^23^, Parkinson^24^, and cardiovascular disease^25^, in which increased expression or destroyed functional integrity can be seen. Moreover, several studies have reported a relationship between HDAC6 and tumours, and HDAC6 is overexpressed in bladder cancer and lung cancer^26^. Regarding tumours of the female reproductive system, HDAC6 is upregulated in oestrogen receptor-positive breast cancer^19^ and ovarian cancer^20^. However, the specific effect of HDAC6 in EC remains unclear. Our research showed that HDAC expression was high in both EC tissues and cell lines and that it promoted tumorigenicity and tumour progression *in vitro*, indicating an important role for HDAC6 in EC.

In 2003, the first HDAC6-specific inhibitor (tubacin) was synthesized, and many inhibitors were subsequently developed. These inhibitors can be classified into four categories: hydroxamates, cyclic peptides, aliphatic acids, and benzamides^27^. Of these inhibitors, tubastatin has the highest activity and selectivity and exhibits the class properties most faithfully^28^, while ricolinostat is most commonly used in clinical trials^29^. In future experiments, we propose to further explore the biological mechanisms of these HDAC6 inhibitors.

Recently, an increasing number of studies have indicated that by targeting oncogenes or antioncogenes, miRNAs could participate in the progression of human cancers. From original articles and literature reviews, Romain *et al*.^9^ suggested that miRNA analysis deserved a role in the evaluation of diagnostic markers and prognostic factors in EC. In addition, several studies^11,12^ have shown that miR-152, miR-200a, miR-200b and miR-429 might play significant roles in EC, and numerous studies have shown that in many cancers, including lung cancer, breast cancer and rhabdomyosarcoma, the expression of miR-206 is decreased^13–15^. However, the precise effect of miR-206 in EC remains unclear. Liu *et al*.^16^ proposed that miR-206 might inhibit the progression of head and neck squamous cell carcinoma by targeting HDAC6. Here, we also showed that miR-206 expression was decreased in EC, which could inhibit its development. Considering that approximately 75–85% of endometrioid EC patients have PTEN mutations and that the PTEN/AKT/mTOR pathway plays a critical role in the development of EC^3^, we sought to determine whether there is a connection between miR-206, HDAC6, PTEN and EC.

Through a search in TargetScan (http://www.targetscan.org/), miR-206 was found to bind directly to the 3′-UTR of HDAC6 mRNA. We then conducted a luciferase reporter assay to validate this prediction. These results indicated that by targeting HDAC6, miR-206 reduced the proliferative and metastatic capacities of EC. To prove this, we performed rescue experiments and found that the inhibitory effect of miR-206 on cell proliferation and invasion was eliminated after co-transfection with the miR-206 mimic and HDAC6 plasmid and that the opposite results occurred after miR-206 silencing. Furthermore, global gene expression analysis was performed, and a heatmap was generated to display the differentially expressed genes after alterations in miR-206 expression in AN3C cells (Fig. 3E). Notably, HDAC6 was among the differentially expressed genes. Subsequent KEGG pathway analysis showed that the AKT signalling pathway might be a relatively critical downstream signalling pathway of miR-206 (Fig. 3F).

Many studies have proven that the PTEN/AKT/mTOR pathway is important in the progression of EC, and signalling through numerous pathways, such as those affecting cellular metabolism, proliferation, migration, apoptosis, and angiogenesis, can be induced by AKT activation^30^. Thus, we selected this pathway for further study, and we found that the expression level of PTEN, the level of p-AKT (S473) and alterations in mTOR accompanied the changes in HDAC6 levels, suggesting that the PTEN/AKT/mTOR pathway is important in EC development (Fig. 6B).

In summary, downregulation of miR-206 and upregulation of HDAC6 were identified in EC and predicted poor prognosis. Additionally, our experimental data showed that miR-206 had a carcinostatic effect on EC by targeting HDAC6 via the PTEN/AKT/mTOR pathway. These findings are expected to benefit EC diagnosis and therapy.

### Supplementary information

Supplementary information.

## Data Availability

The online data used to support the findings of this study are included within the article, and the experimental data used to support the findings are available from the corresponding author upon request.
