# Author Correction: HDAC6, modulated by miR-206, promotes endometrial cancer progression through the PTEN/AKT/mTOR pathway

**DOI:** 10.1038/s41598-024-64863-2

**Published:** 2024-07-12

**Authors:** Yawen Zheng, Xiaohui Yang, Chunyan Wang, Shuo Zhang, Zhiling Wang, Meng Li, Yuanjian Wang, Xiaojie Wang, Xingsheng Yang

**Affiliations:** 1 Department of Obstetrics and Gynecology, Qilu Hospital of Shandong University, Jinan, Shandong China; 2 West China School of Medicine, Sichuan University, Chengdu, Sichuan China; 3 Department of Dermatology, Peking University People’s Hospital, Beijing, China

Correction to: *Scientific Reports* 10.1038/s41598-020-60271-4, published online 27 February 2020

The original Article contained an error in Figure 5. Due to a mistake in figure assembly, the panel Ishikawa^miR-206+/HDAC6+^ 24h was duplicated from the panel Ishikawa^miR-206+^ 48h.

The original Figure 5 and accompanying legend appear below as Figure 1.

**Figure 1:**
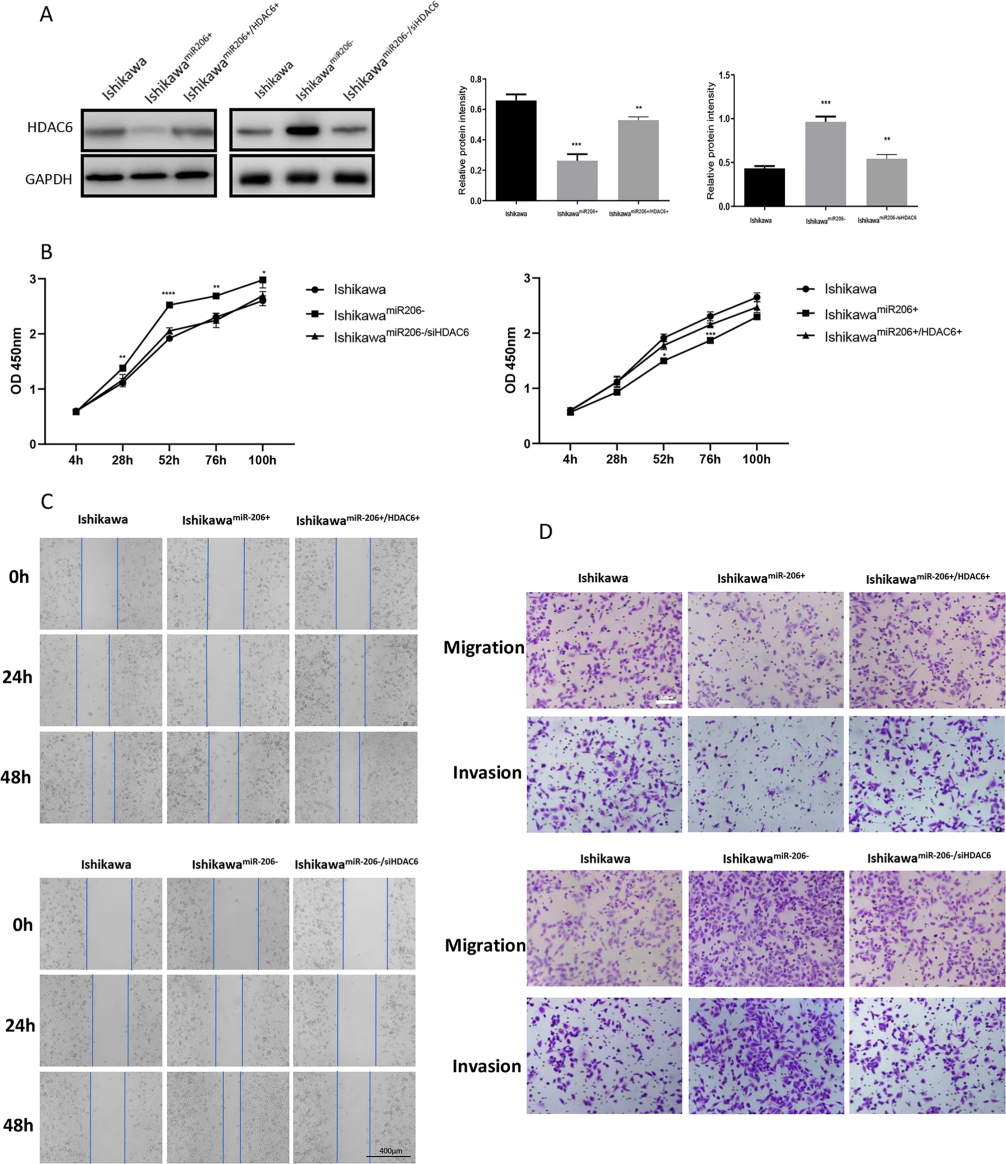
HDAC6 can reverse the effect of miR-206 on EC cells. (**A**) Western blotting showed that HDAC6 protein levels were reversed in Ishikawa cells after co-transfection with the miR-206 mimic and HDAC6 plasmid. (**B**) CCK-8 assay after co-transfection of Ishikawa cells. (**C**) Wound healing assay after co-transfection of Ishikawa cells. Scale bar, 400 μm. (**D**) Transwell migration and invasion assays after co-transfection of Ishikawa cells. Scale bar, 100 μm. ***P < 0.001, ****P < 0.0001.

The original Article has been corrected.

