# Supplementary material for: HDAC6, modulated by miR-206, promotes endometrial cancer progression through the PTEN/AKT/mTOR pathway
Source: Sci Rep. 2020 Feb 27;10:3576. doi: 10.1038/s41598-020-60271-4 (PMC7046652; doi:10.1038/s41598-020-60271-4)
Supplement: Supplementary file 1 — Supplementary information. [file 41598_2020_60271_MOESM1_ESM.pdf]

HDAC6, modulated by miR-206, promotes endometrial cancer progression through PTEN/AKT/mTOR pathway

Yawen Zheng[1], Xiaohui Yang[1], Chunyan Wang[1], Shuo Zhang [1], Zhiling Wang[1], Meng Li[1], Yuanjian Wang[2], Xiaojie Wang[3], Xingsheng Yang*

Author affiliations:

[1] Department of Obstetrics and Gynecology, Qilu Hospital of Shandong University, Jinan, Shandong, China;

[2] West China School of Medicine , Sichuan University, Chengdu, Sichuan, China;

[3] Department of dermatology, Peking University People's Hospital, Beijing, China;

*Department of Obstetrics and Gynecology, Qilu Hospital of Shandong University, Jinan, Shandong, China. Electronic address: xingshengyang@sdu.edu.cn.

Supplementary Figure 1

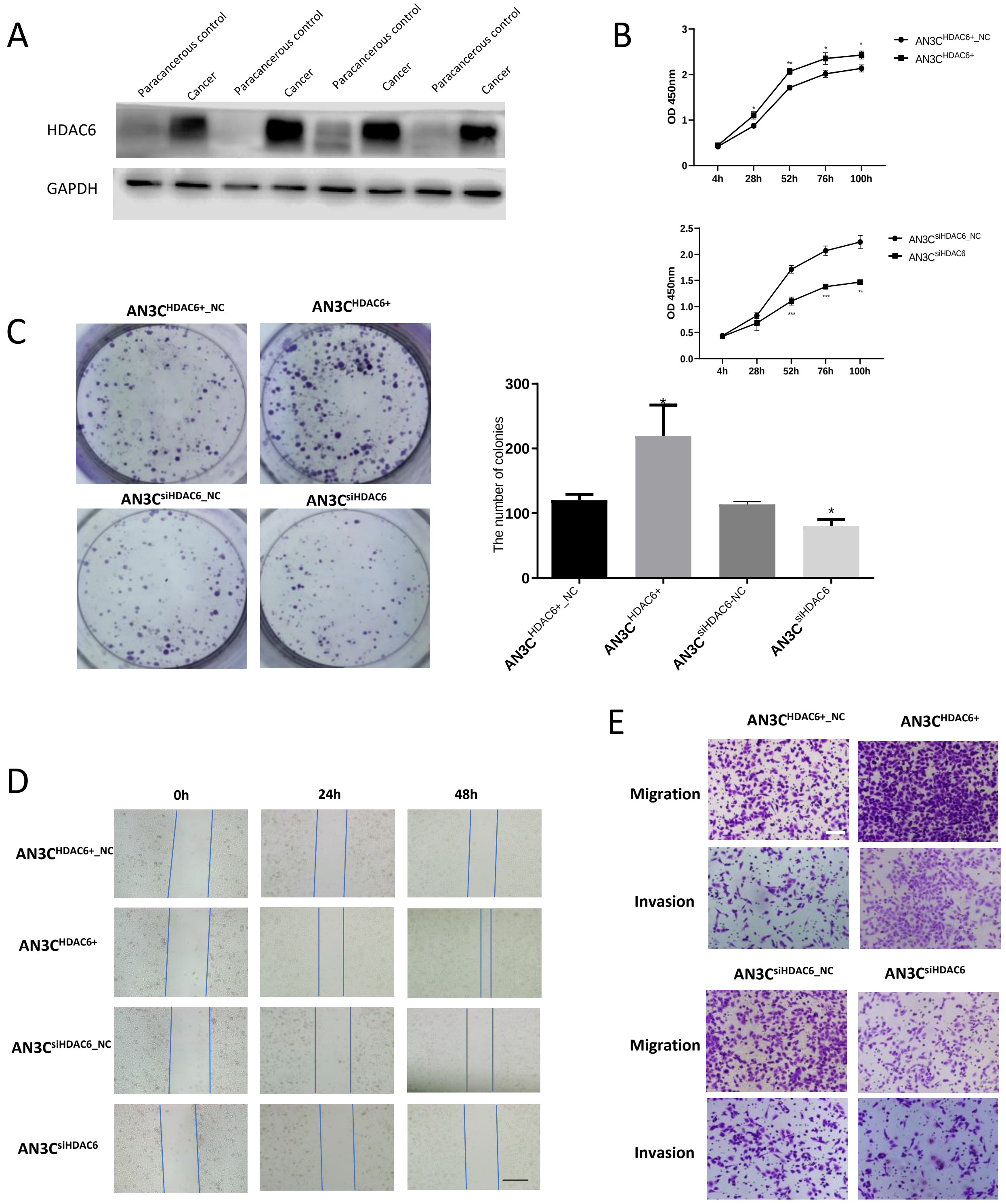

Supplementary Figure 1 (A) Western blot showed that cancer tissues expressed more HDAC6 than paracancerous controls. (B) CCK-8 assay after overexpression and knockdown of HDAC6 in AN3C cells. (C) Colony formation assay after overexpression and knockdown of HDAC6 in AN3C cells. (D) Scratch wound assay after overexpression and knockdown of HDAC6 in AN3C cells. Scale bar, 400μm. (E) Transwell migration and invasion assay after overexpression and knockdown of HDAC6 in Ishikawa cells. Scale bar, 100μm. *P<0.05, **P<0.01.

Supplementary Figure 2

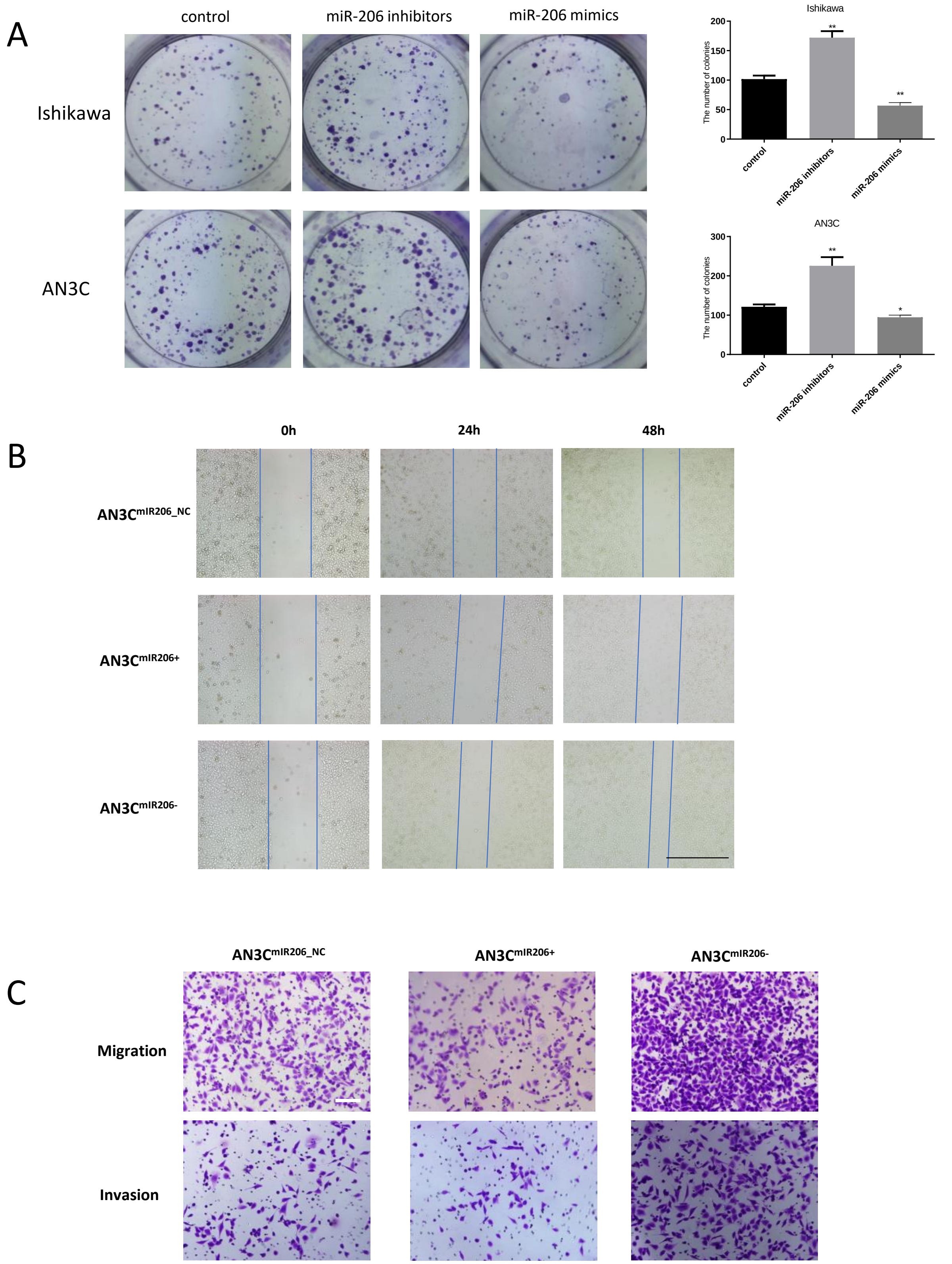

Supplementary Figure 2 (A) Colony formation assay after overexpression and knockdown of miR-206 in Ishikawa and AN3C cells. (B) Scratch wound assay after overexpression and knockdown of miR-206 in AN3C cells. Scale bar, 400μm. (C) Transwell migration and invasion assay after overexpression and knockdown of miR-206 in Ishikawa cells. Scale bar, 100μm. **P*<0.05, ***P*<0.01.

Supplementary Table

Gene Name	Ensembl	baseMean	log2FoldChange	lfcSE	stat	pvalue	padj
KRT7	ENSG00000135480	727.493309	-0.9900722	0.243662	-4.0632967	4.84E-05	0.041654
LCP1	ENSG00000136167	1675.559346	-0.7783319	0.196606	-3.9588365	7.53E-05	0.055576
HDAC6	ENSG00000094631	1932.834987	-0.7253764	0.188277	-3.8526997	0.000116823	0.072411
EFNA1	ENSG00000169242	3295.016098	-0.7952309	0.173063	-4.5950473	4.33E-06	0.009733
C3	ENSG00000125730	952.6943918	-1.0466812	0.227951	-4.5916975	4.40E-06	0.009733
PXDN	ENSG00000130508	121.3439916	-2.0858284	0.508512	-4.101828	4.10E-05	0.037363
INSIG1	ENSG00000186480	7746.978838	-0.7651794	0.17028	-4.4936438	7.00E-06	0.012037
CCL2	ENSG00000108691	351.0766633	-1.2219424	0.306344	-3.9887913	6.64E-05	0.051455
IL6	ENSG00000136244	693.2015904	-1.0794603	0.24141	-4.4714783	7.77E-06	0.012037
FN1	ENSG00000115414	17319.59958	-0.666115	0.156567	-4.2545037	2.10E-05	0.022885
ALCAM	ENSG00000170017	527.5250384	-1.1329684	0.288882	-3.9219058	8.79E-05	0.061879
SLC44A1	ENSG00000070214	644.3436071	-0.9593053	0.246636	-3.8895589	0.000100427	0.064842
CXCL8	ENSG00000169429	1729.333596	-1.1876301	0.218185	-5.4432347	5.23E-08	0.000405
ID1	ENSG00000125968	7024.5376	0.83744579	0.165431	5.0622089	4.14E-07	0.001605
FOS	ENSG00000170345	6070.50122	0.7160539	0.163402	4.38216994	1.18E-05	0.015174
NR4A1	ENSG00000123358	2757.670816	0.7596671	0.178739	4.25014945	2.14E-05	0.022885
FOSB	ENSG00000125740	3379.56025	1.00997711	0.172679	5.8488585	4.95E-09	7.67E-05
C4orf48	ENSG00000243449	448.3738962	1.54762465	0.317724	4.87097346	1.11E-06	0.003442
CRLS1	ENSG00000088766	314.6373119	1.49973438	0.353543	4.24200817	2.22E-05	0.022885
C1QBP	ENSG00000108561	11775.24762	0.64720776	0.161315	4.0120798	6.02E-05	0.049087
RAPGEF3	ENSG00000079337	2020.971092	0.82651863	0.186996	4.41996967	9.87E-06	0.013906
KLF2	ENSG00000127528	1933.131817	0.87179561	0.192435	4.53032926	5.89E-06	0.011407
ANKRD1	ENSG00000148677	8919.875478	0.60866518	0.161518	3.76839397	0.000164301	0.097924
FOSL1	ENSG00000175592	2510.472468	0.76835047	0.184254	4.17006873	3.05E-05	0.029492
NDUFAF8	ENSG00000224877	1273.97307	0.80578758	0.206734	3.89769794	9.71E-05	0.064842

Supplementary Table Top 25 differentially expressed mRNAs in miR-206 inhibitor/mimics-transfected AN3C cells vs control.
